# Descriptions of *Mikrocytos veneroïdes* n. sp. and *Mikrocytos donaxi* n. sp. (Ascetosporea: Mikrocytida: Mikrocytiidae), detected during important mortality events of the wedge clam *Donax trunculus* Linnaeus (Veneroida: Donacidae), in France between 2008 and 2011

**DOI:** 10.1186/s13071-018-2692-0

**Published:** 2018-03-02

**Authors:** Céline Garcia, Christophe Haond, Bruno Chollet, Mirella Nerac, Emmanuelle Omnes, Jean-Pierre Joly, Christine Dubreuil, Delphine Serpin, Aimé Langlade, Dominique Le Gal, Aouregan Terre-Terrillon, Olivier Courtois, Benjamin Guichard, Isabelle Arzul

**Affiliations:** 1 Ifremer, RBE-SG2M-LGPMM, Station de La Tremblade, Avenue de Mus de Loup, F-17390 La Tremblade, France; 2 Ifremer, ODE-LITTORAL-LERMPL, Station de la Trinité sur Mer, 12, rue des Résistants, F-56470 La Trinité sur Mer, France; 3 Ifremer, ODE-LITTORAL-LERBO, Station de Concarneau, Place de la Croix, F-29185 Concarneau, France; 4 Ifremer, ODE-LITTORAL-LERPC, Station de La Tremblade, Avenue de Mus de Loup, F-17390 La Tremblade, France

**Keywords:** *Mikrocytos veneroïdes * n. sp., *Mikrocytos donaxi * n. sp., *Donax trunculus*, Mortality, Microcell parasite

## Abstract

**Background:**

Microcell parasites are small intracellular protozoans mostly detected in molluscs and can be associated with mortalities. In 2010 and 2011, strong increases in mortality events were reported in different wild beds of the wedge clam *Donax trunculus* Linnaeus, along the Atlantic coast of France and the presence of potential pathogens, including microcells, was investigated.

**Methods:**

Clams collected in different beds showing mortality were examined by histology. Based on histological observations, confirmatory analyses were carried out, including transmission electron microscopy (TEM) and molecular characterization.

**Results:**

Histological analyses revealed the presence of small protozoans similar to microcell parasites in different tissues of *Donax trunculus*, particularly in muscular and connective tissues. TEM examination confirmed the intracellular localization of the protozoans. Moreover, the lack of haplosporosomes and mitochondria suggested that the observed parasites belong to the genus *Mikrocytos* Farley, Wolf & Elston, 1988. *Mikrocytos* genus-specific PCR and in situ hybridization results supported the microscopic observations. Sequence fragments of the *18S* rRNA gene shared 75–83% identity with the different *Mikrocytos* spp. described previously, including *Mikrocytos mackini* Farley, Wolf & Elston, 1988 and *M. boweri* Abbott, Meyer, Lowe, Kim & Johnson, 2014. Phylogenetic analyses confirmed that the microcell parasites observed in *Donax trunculus* in France belong to the genus *Mikrocytos* and suggest the existence of two distinct species.

**Conclusions:**

Based on morphological, ultrastructural, molecular data and host information, the two microcell parasites detected in *Donax trunculus* belong to the genus *Mikrocytos* and are distinct from previously described members of this genus. This is the first report of *Mikrocytos* spp. found in France and infecting the clam *Donax trunculus*. *Mikrocytos veneroïdes* n. sp. was detected in different wild beds and *Mikrocytos donaxi* n. sp. was detected only in Audierne Bay.

**Electronic supplementary material:**

The online version of this article (10.1186/s13071-018-2692-0) contains supplementary material, which is available to authorized users.

## Background

Microcell parasites are small intracellular protozoans (2–3 μm) that are essentially mollusc parasites within two genera, *Bonamia* Pichot, Comps, Tigé, Grizel & Rabouin, 1980 and *Mikrocytos* Farley, Wolf & Elston, 1988*.* The genus *Bonamia* is classified in the order of Haplosporidia Caullery & Mesnil, 1899 [1] and all protozoans of this genus are parasites of oysters, such as *Bonamia ostreae* Pichot, Comps, Tigé, Grizel & Rabouin, 1980, a parasite of European flat oyster *Ostrea edulis* Linnaeus, 1758 [2]. For a long time, the relationship of the genus *Mikrocytos* to other known protist taxa was not known [3]. Recently, it was affiliated with the supergroup Rhizaria Cavalier-Smith, 2002 [4] and placed in a new taxonomic order, Mikrocytida Hartikainen, Stentiford, Bateman, Berney, Feist, Longshaw, Okamura, Stone, Ward, Wood & Bass, 2014, a sister group to the taxonomic order of Haplosporidia [5, 6]. Until 2013, only one species, *Mikrocytos mackini* Farley, Wolf & Elston, 1988, had been described in the genus, since *M. roughleyi* Farley, Wolf & Elston 1988 was placed in the genus *Bonamia* [7]. The protozoan *Mikrocytos mackini* was first reported in 1961 [8] and was validly described and named in 1988 [9]. This parasite was responsible for Denman Island disease in the wild and cultured Pacific oyster *Crassostrea gigas* Thunberg, 1793 in British Columbia, Canada, where it induced severe oyster mortalities [8]. It could naturally affect other oysters, such as the Olympia oyster *Ostrea lurida* Carpenter, 1864 [10] or the Kumamoto oyster *Crassostrea sikamea* Amemiya, 1928 [11]. Since its detection, the range extension of this parasite has been limited to the west coast of North America, in Canada and the USA [9, 11–13]; it has never been detected in Europe. Because of its potential impact on oyster farming, this parasite is regulated in Europe and surveillance is implemented in different European countries in order to prevent its introduction.

Recently, two new species of *Mikrocytos* have been described: *M. boweri* Abbott, Meyer, Lowe, Kim & Johnson, 2014 in *Ostrea lurida* from British Columbia, Canada [14] and *M. mimicus* Hartikainen, Stentiford, Bateman, Berney, Feist, Longshaw, Okamura, Stone, Ward, Wood & Bass, 2014 in *Crassostrea gigas* from Brancaster, UK [5]. Other *Mikrocytos*-like protists have been reported in different areas, although none of these parasites has yet been formally named. Indeed, three different isolates with a strong similarity in the *18S* rDNA fragment were hypothesized to be members of *Mikrocytos*, but distinct from *M. mackini*: one in *Ostrea edulis* from Atlantic Canada that had been imported into France for research purposes [15], one in *Crassostrea gigas* from China [16] and one in *C. gigas* on the west coast of Canada [17]. Moreover, another species of *Mikrocytos* sharing a homologous *18S* fragment with *M. mimicus* was described in Spain in the Manila clam *Ruditapes philippinarum* Adams & Reeve, 1850 [18]. In the last few years, detection of parasites belonging to this genus has increased, along with their geographical distribution specifically with the first descriptions of these parasites in Europe [5, 18].

In France, although the mollusc production relies mainly on the Pacific oyster *Crassostrea gigas*, species of *Mikrocytos* including *M. mackini* have never been detected [19]. In the last few years, *C. gigas* production has decreased in France, due to different infectious agents, such as the virus OsHV-1 Davison, Trus, Cheng, Steven, Watson, Cunningham, LeDeuff & Renault 2005 and bacteria belonging to the genus *Vibrio* Pacini, 1854 [20–22] but no regulated parasites, including *M. mackini*, were detected. In addition to *C. gigas*, other bivalve molluscs have been cultivated or harvested, including the wedge clam *Donax trunculus* Linnaeus, 1758. This bivalve is mainly distributed on exposed beaches, in the soft-bottom intertidal areas of numerous bays and estuaries in western and southern Europe [23]. In France, commercial exploitation (around 1000 tons/year) is based on natural populations distributed along both the Atlantic and Mediterranean coasts [24]. The production has been stable for several years and is controlled by a limited number of fishing licences, but little information is available concerning the factors influencing its survival. Some parasites have been reported in this bivalve, such as the trematodes *Bacciger bacciger* (Rudolphi, 1819) Nicoll, 1914 [25], *Parvatrema strigatum* (Lebour, 1908) Bartoli, 1983 [26] and *Postmonorchis* sp. [27], some rickettsia-like organisms [28] and coccidian-like organisms [29]. However, their impact on *Donax trunculus* is not really known. Recently, the impact of the trematode *B. bacciger* on this bivalve species was investigated and it was shown that it could have a deleterious effect [30]. In France, this bivalve is not specifically monitored unless mortalities are reported. Thus, in 2010 and 2011, severe mortality events occurred on different wedge clam beds in France and analyses revealed the presence of microcell parasites belonging to *Mikrocytos* in the different sampled areas. This was the first time that *Mikrocytos* parasites were detected in France and the first occurrence of these parasites in this bivalve species. With this detection, several questions arose including: what is the relative taxonomic position of these parasites? Are they new species or known species? What is their role in observed mortality? In order to answer these questions, we characterized these parasites on the basis of morphological, ultrastructural and molecular criteria, and also by including host information as suggested by Abbott & Meyer [31]. The data obtained allowed us to provide a detailed description of two new species, *Mikrocytos veneroïdes* n. sp. and *M. donaxi* n. sp., detected during mortality events of the wedge clam *Donax trunculus* in France. We also discussed about the simultaneous apparition of these two different parasites in very close locations and their potential role in *D. trunculus* mortality.

## Methods

### Sample collection

**Fig. 1:**
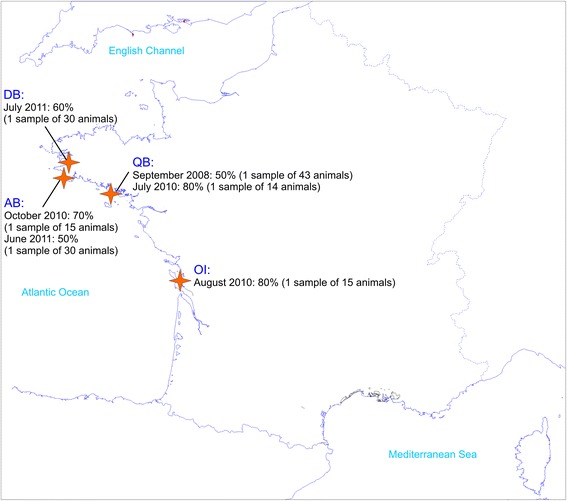
Sampling sites of *Donax trunculus*, mortality rate estimation and collected individuals along the Atlantic coast of France. *Abbreviations*: AB, Audierne Bay; DB, Douarnenez Bay; QB, Quiberon Bay; OI, Oléron Island

Samples of the wedge clam *Donax trunculus* were collected in 2010 and 2011 in the context of increased mollusc mortality in different wild beds of the French Atlantic coast (Fig. 1). Mortality events were reported by fishermen to the competent authority and the national network for surveillance and monitoring of mollusc health, called REPAMO (REseau de PAthologie des MOllusques), who recorded mortality cases and performed analysis on representative samples associated with the mortality cases. For each mortality case, data were collected from fishermen by face to face interviews using a questionnaire that helped to explore the different factors which could be involved in the mortality events. In 2010, 15 individuals per sample were collected in three beds and in 2011, 30 individuals of two beds were sampled (Fig. 1). Each collected individual was examined for macroscopic information and processed using histological and molecular analyses. For 6 individuals sampled in 2011, tissue samples were analysed by transmission electron microscopy (TEM).

In addition, archived material of *D. trunculus* collected during a mortality event in 2008 in Quiberon Bay (Fig. 1) was also tested with molecular biology techniques (43 individuals) and TEM (3 individuals), after histological observations showed the presence of microcell parasites in some individuals. In 2008, no positive result was obtained with in situ hybridization using probes specific to the genus *Bonamia* and *Mikrocytos mackini*.

### Histology and in situ hybridization (ISH)

After 48 h in Davidson’s fixative, tissues (mantle, digestive gland, gills, foot, adductor muscle and kidney) were maintained in 70% ethanol until dehydrated and embedded in paraffin for histology according to standard procedures. Sections of 2–3 μm thickness were stained by hematoxylin and eosin.

Eleven individuals (6 from Oléron Island 2010, 1 from Audierne Bay 2010 and 4 from Quiberon Bay 2008) were processed for ISH testing. The *Mikrocytos* genus-specific primer pairs Msp 443F and Msp746R developed by Gagné et al. [15] were used to produce a digoxigenin-labelled probe by PCR. In situ hybridisation was performed following procedures previously described by Arzul et al. [32], except that tissue sections were 7 μm thick and a pre-hybridization step was carried out for 30 min at 42 °C in a humid chamber. Negative controls included samples without digoxigenin-labelled probe in the hybridization mixture or without antibodies during the revelation step. Positive control consisted of *Crassostrea gigas* infected with *Mikrocytos mackini* originating from Canada (kindly provided by G. Meyer).

### Transmission electron microscopy (TEM)

Small pieces of mantle and adductor muscle (1–2 mm) of nine individuals (three each from Quiberon Bay, Audierne Bay and Douarnenez Bay) were fixed in 3% glutaraldehyde. The ultra-thin sections were obtained using the procedures described by Garcia et al. [33]. Copper EM grids were stained with uracyl acetate/lead citrate. The sections were examined using an electron microscope (JEM 1011, JEOL) at 80 kV.

### DNA extraction

Genomic DNA from all sampled individuals was extracted from excised mantle, adductor muscle and gills of *D. trunculus*. The protocol used for DNA extraction was that published by Winnepenninckx et al. [34] with the modifications published by Lopez-Flores et al. [35]. DNA was eluted and resuspended in a final volume of 50 μl sterile deionised water and then diluted to a final concentration of 100 ng/μl.

### Confirmation of identity of the wedge clams

Identification of *Donax* Linnaeus, 1758 species for all sampled individuals was performed using the PCR protocol described by Pereira et al. [36] for the amplification of the *5S* region using primer pair 5SF/5SR. The length of fragments was determined by comparison with Smart Ladder SF 100–1000 bp marker (Eurogentec, Liège, Belgium) after electrophoresis on a 1.5% agarose gel. The size of expected PCR products was 275–300 bp.

### Microcell characterization: PCR, cloning and sequencing

PCR assays on *Donax trunculus* (Table 1) were done initially with the *Bonamia* genus-specific primer pair Bo [37] and *Mikrocytos* genus-specific primer pair MM [3]. Subsequently, specific *Mikrocytos*-like primer pair MM-like and another *Mikrocytos* genus-specific primer pair Msp targeting the *18S* region were used [15].

**Table 1:** Histological and microcell PCR results obtained for *Donax trunculus* samples for each location and year included in the present study

Year		2010			2011		2008
Area		Quiberon	Oléron	Audierne	Audierne	Douarnenez	Quiberon
Sample reference		10_138	10_143	10_171	11_089	11_118	08_131
No. of individuals analysed		14	15	15	30	30	43
Histology, *n* (%) infected individuals	Microcell parasites	6 (42.9)	12 (80.0)	2 (13.3)	22 (73.3)	20 (66.7)	14 (32.6)
	Coccidia-like	0	7 (46.7)	0	7 (23.3)	22 (73.3)	9 (21.0)
	Rickettsia-like	0	5 (33.3)	7 (46.7)	4 (13.3)	2 (6.7)	3 (7.0)
	Gregarine spores	3 (21.4)	8 (53.3)	13 (86.7)	18 (60.0)	25 (83.3)	17 (39.6)
	Trematodes	0	0	0	0	6 (20.0)	1 (2.3)
PCR for microcell detection, *n* (%) infected individuals	Bo primers	0	0	0	0	0	0
	MM primers	0	0	0	5 (13.7)	0	0
	MM-like primers	0	0	0	nd	nd	nd
	Msp primers	7 (50.0)	6 (40.0)	3 (20.0)	20 (66.7)	24 (80.0)	6 (13.9)

*Abbreviation*: *nd* not done

In order to amplify a longer *18S* fragment, 13 different primer pair associations using previous primers and primers developed by Abbott et al. [17] and Hartikainen et al. [5] (mik451F, mik1511R, Mm18S_120F, Mm18S_1403R, 18S_EUK1776-R, Mm18S_SF1, Mm18S_SF3, Mm18S_1450R, Mm18S_1128F, pro28SR) were used on twelve individuals, six each from Audierne Bay and Douarnenez Bay presenting microcell parasites in histological analyses. In addition, DNA extracted from six individuals, three each from Audierne Bay and Douarnenez Bay were amplified using Mm18S_1435F-Mm28SR1 primers targeting the *ITS1-5.8S-ITS2* region of these microcell parasites [17]. All PCR reactions were performed using Go Taq polymerase (Promega, Fitchburg, Wisconsin, USA) in a final volume of 50 μl: 1 μl DNA (50–100 ng/μl) was added to 49 μl of the PCR mix. Mix composition and thermal cycling conditions were as described in [3, 15, 17, 37], according the primer pair used. Negative controls consisting of water were included for every 10 tested samples in order to check for potential contamination. A positive control was performed on DNA extracted from *Crassostrea gigas* tissues infected with *Mikrocytos mackini* originating from Canada (kindly provided by G. Meyer).

In order to detect potential co-infection, fifteen PCR products (three from each location and three from archived material) targeting the *18S* region were cloned using the TOPO TA cloning kit (Invitrogen, Waltham, Massachusetts, USA), according to the manufacturer’s recommendations, and positive clones were then selected for plasmid DNA purification by FastPlasmid® Min (Eppendorf, Hamburg, Germany).

Plasmid DNA suspensions and PCR products targeting the *ITS1-5.8S-ITS2* region were bi-directionally sequenced using the Big Dye V3 sequencing kit (Applied Biosystem, Waltham, Massachusetts, USA). The resulting sequences were deposited in the GenBank database under accession numbers KY923792–KY923807 for partial *18S* region and KY923808–KY923809 for *ITS1-5.8S-ITS2* region.

### Phylogenetic analyses

Sequences obtained were compared with those in the GenBank database using the BLAST algorithm [38]. Available ITS and *18S* gene sequences from *Mikrocytos* spp. and *Paramikrocytos canceri* Hartikainen, Stentiford, Bateman, Berney, Feist, Longshaw, Okamura, Stone, Ward, Wood & Bass, 2014 were downloaded from GenBank and included in the phylogenetic analyses together with the sequences obtained in the present study. *18S* gene sequences from GenBank included: *Mikrocytos mackini* (HM563060.1, AF477623.1), *Mikrocytos* sp. from British Columbia (HM563061.1, DQ237912.1), *Mikrocytos* sp. from Spain (KF548052.1, KF548051.1), *Mikrocytos boweri* (KF297353.1), *Mikrocytos mimicus* (KP164508.1) and *Paramikrocytos canceri* (KJ150292). rDNA *ITS* sequences from GenBank included: *Mikrocytos mackini* (HM563060.1), *Mikrocytos* sp. from Spain (KF5480485), and *Mikrocytos boweri* (KF297352.1). Alignments were performed using ClustalW [39] in MEGA 6 with default parameters. The average length of the 25 *18S* gene sequences in the final alignment was 328 bp. For the nine *ITS* sequences it was 535 bp. The alignments were manually checked for alignment gaps and missing data in nucleotide positions.

Evolutionary divergence over sequence pairs between groups of sequences was estimated using the Maximum Composite Likelihood model [40]. The rate variation among sites was modelled with a gamma distribution (shape parameter = 1). Differences in the composition bias among sequences were considered in evolutionary comparisons [41]. For *18S* gene sequences, the analysis involved 25 nucleotide sequences. All positions containing gaps and missing data were eliminated, resulting in a total of 253 positions in the final dataset. For *ITS1-5.8S-ITS2* sequences, the analysis involved 9 nucleotide sequences. Analysis was carried out on the complete array *ITS1-5.8S-ITS2* and on each gene. All positions containing gaps and missing data were eliminated, leaving a total of 344, 82, 118 and 142 positions in the final dataset for *ITS1-5.8S-ITS2*, *ITS1*, *5.8S* and *ITS2*, respectively. Evolutionary analyses were conducted using MEGA 6 [38].

Prior to the phylogenetic analysis of sequences, the program jModelTest 0.1.1 [42] was used to select the best fitting substitution model according to the corrected Akaike information criterion (AIC) [43]. A total of 88 candidate models, including models with equal/unequal base frequencies, with/without a proportion of invariable sites (+I), and with/without rate variation among sites (+G) were tested. For *18S* gene sequences, the best-fit model of nucleotide substitution was the TPM3uf + G with unequal base frequencies and AC = CG; AT = GT; AG = CT as substitution rates. However, the weight for this model was not high (0.1278), indicating model selection uncertainty. The 99% cumulative weight interval included the most complex model evaluated (GTR + G + I). Tree topology was therefore inferred based on a Bayesian approach using MrBayes v 3.1.2 [44] and implementing the GTR + I + G model of nucleotide substitution. For *ITS1*, *5.8S* and *ITS2* sequences, the best-fit models of nucleotide substitution were TPM2uf + G, TPM3uf and TrN + G, respectively. However, similarly for the *18S* gene sequences, the 99% cumulative weight interval included the most complex model evaluated (GTR + G + I) which was therefore implemented using MrBayes v 3.1.2.

### Other pathogen detection

Virus OsHV-1 and bacteria belonging to the genus *Vibrio*, which are involved in different mortality events of molluscs, were screened in all 2010 and 2011 samples. Detection and quantification of OsHV-1 was performed using the real time PCR technique developed by Pepin et al. [45] and the protocol described in [46]. In these quantitative PCR tests, the primer pairs used to detect viral DNA were those described by Webb et al. [47], targeting the OsHV-1 DNA polymerase. For detection and quantification of bacteria, pieces of gills and mantle of five individuals per sample were individually crushed in 150 μl sterile seawater. Then, 100 μl of supernatant was diluted 100-fold and 10,000-fold and each dilution was spread onto Marine agar (Conda, Madrid, Spain) in Petri dishes, which were incubated for 72 h at 20 °C. Colonies were enumerated and the bacterial colonies found in abundance were picked up and tested by duplex real time PCR for the detection of *Vibrio aestuarianus* Tison & Seidler, 1983 and bacteria of the *Splendidus* clade following the protocol described by Saulnier et al. [48].

## Results

### Description of *Donax* mortality events

In 2010, important mortality events of *Donax trunculus* were reported in three different wild beds: Quiberon Bay, Oléron Island and Audierne Bay. The mortality rate was estimated between 70 and 80% according to the site. In 2011, new increased mortality events occurred in two locations: Audierne Bay (same site as 2010) and Douarnenez Bay. The mortality rate was also high (Fig. 1). In all cases, the mortality was uniform, massive, sudden and concerned *Donax trunculus* of all ages; no other species were affected by these mortality events. The mortality occurred during summer and the beginning of autumn (no mortality was reported at the other periods of the year) and mortality events lasted from 2 weeks to 1 month, according to fishermen. No specific environmental event, such as algal bloom, was reported in 2010 and 2011. Similar observations were made during the mortality event occurring in September 2008 in Quiberon Bay.

The sampled individuals were mainly moribund, except for the Audierne 2010 sample where most individuals were alive; this sample was collected after the mortality event. Moribund animals were characterized by a very slow closing of their valves and a limited intra-palleal fluid. Individuals showed no specific macroscopic signs and in particular, no signs such as pustules, conchyolin deposit or necrosis and degradation of the hinge were observed.

### Identity of the wedge clams

Molecular identification verified that all *Donax* species analysed were *Donax trunculus*. All individuals from all locations shared the same size of the PCR product (300 bp).

### General observations on wedge clam pathogens

**Fig. 2:**
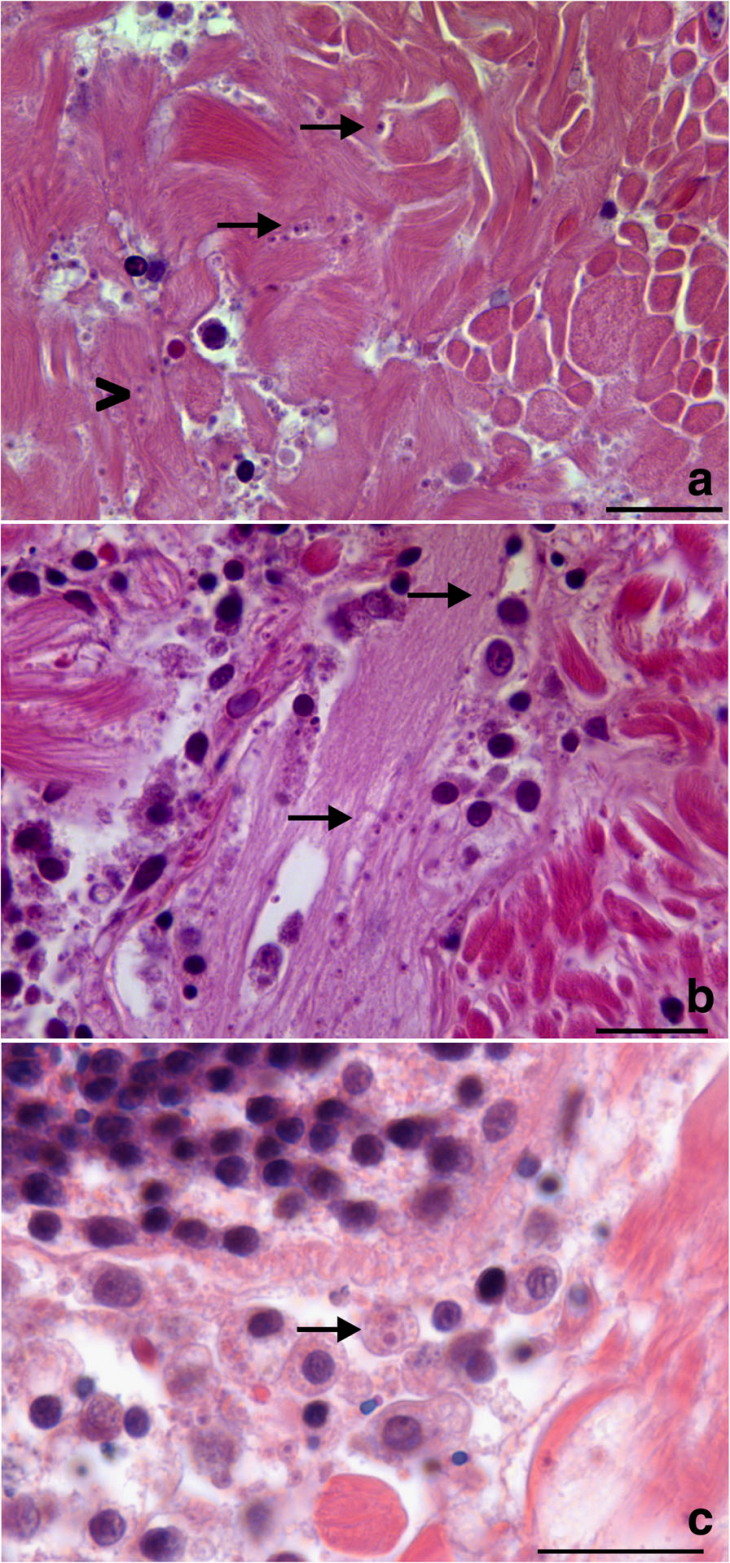
Histological haematoxylin eosin tissue sections showing *Mikrocytos veneroïdes* n. sp. parasites in different tissues of *Donax trunculus* from Oléron Island. **a** Parasites in the adductor muscle. Note the extracellular (arrows) or intracellular (arrowhead) position of microcell parasites. **b** Parasite cell (arrows) in the neuronal ganglion. **c** Two parasites (arrow) inside the cytoplasm of a haemocyte. *Scale-bars*: 20 μm

**Fig. 3:**
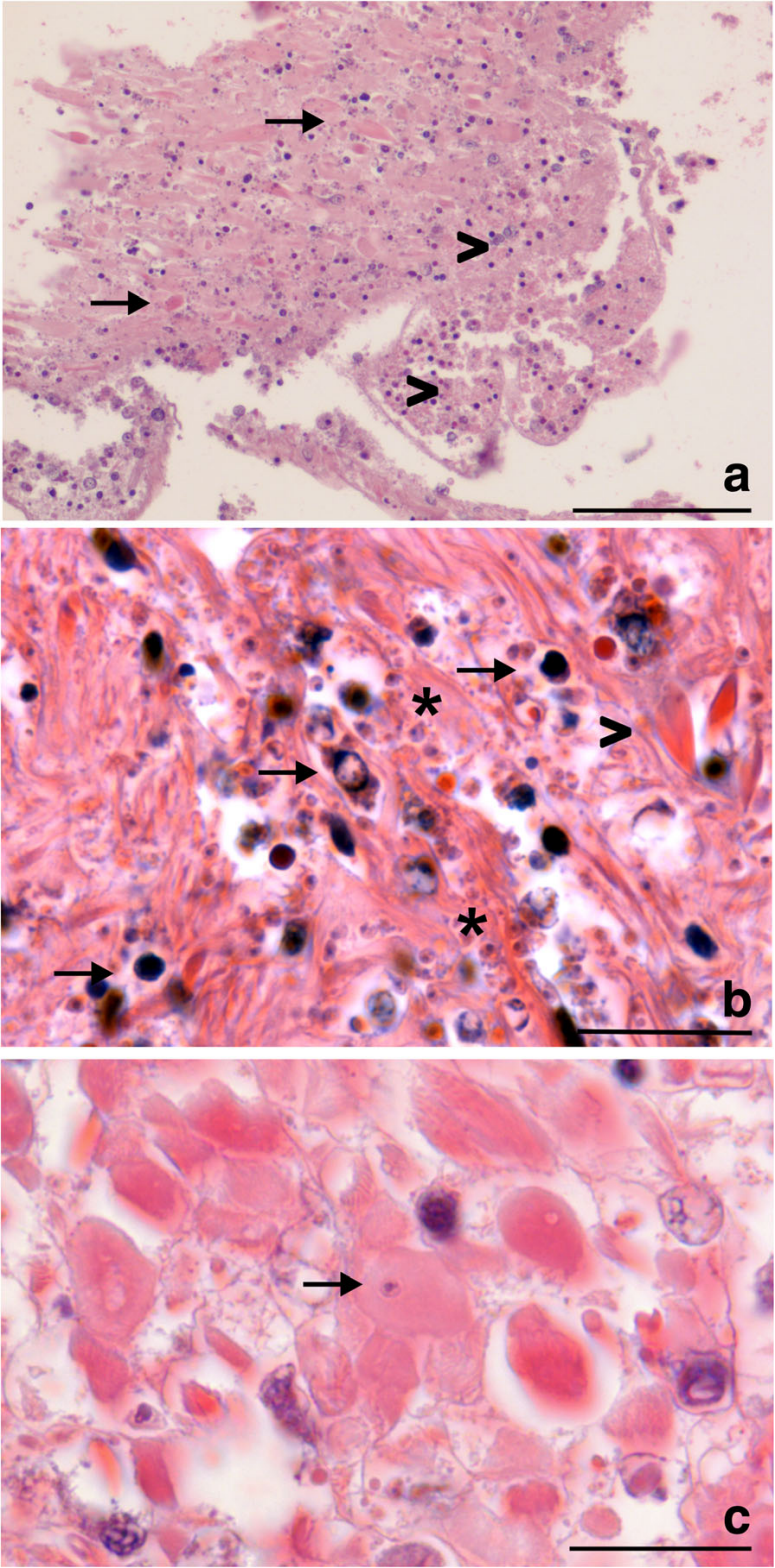
Histological haematoxylin eosin tissue sections showing mantle lesions of *Donax trunculus* infected with *Mikrocytos donaxi* n. sp. parasites from Audierne Bay. **a** Diffuse necrosis of muscular fibres (arrows) and connective tissue (arrowheads) of the mantle infected with microcell parasites **b** Degenerated haemocytes (arrows) and muscular cell necrosis (arrowhead) associated with microcell parasites (asterisks). **c** *Mikrocytos donaxi* n. sp. inside a myocyte (arrow). *Scale-bars*: **a**, 100 μm; **b**, **c**, 20 μm

**Fig. 4:**
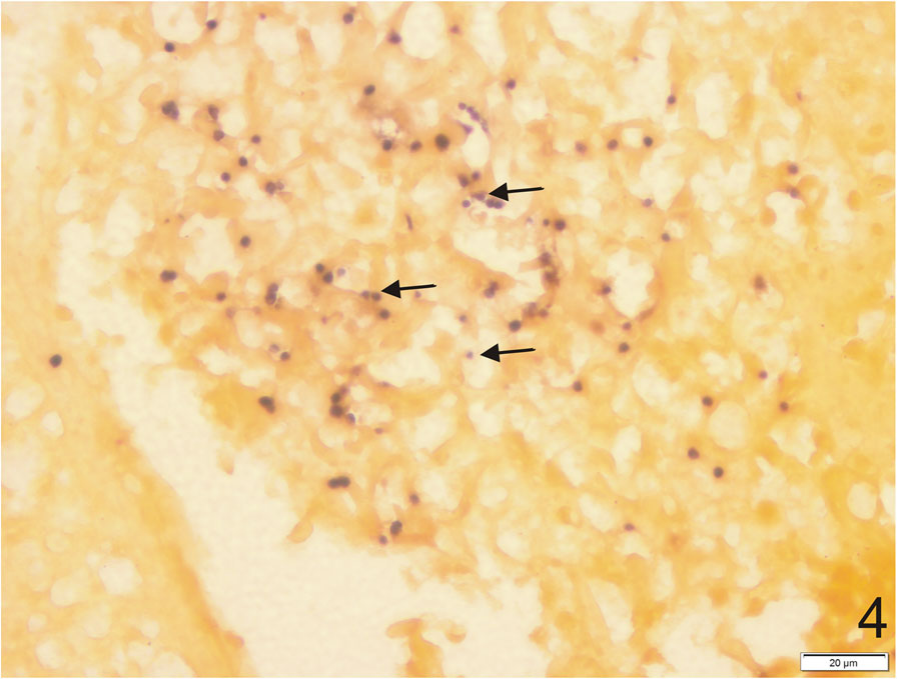
Histological section of *Donax trunculus* mantle showing hybridization of the *Mikrocytos* Msp probe with microcell cells (arrows). *Scale-bar*: 20 μm

In all sites sampled, histological analyses revealed the presence of microcell parasites, measuring 1.79–2.15 μm (Tables 1 and 2) and distributed throughout the clam tissues (Figs. 2 and 3). In most cases, the infection was focal and low for any given organ, but infected individuals presented several focal infections distributed in different organs. In all cases, no major haemocyte infiltration or abscess was found associated with the presence of these microcell parasites (Fig. 3a). However, the parasites were associated with adjacent tissue necrosis (Fig. 3b). ISH was used to confirm the presence of the microcells. Eleven tested individuals showed specific labelling of parasite cells with the Msp probe. Vesicular connective tissue and muscular fibres of all organs appeared to be infected by the parasites (Fig. 4). The infection intensity seemed higher than in the histological observations, with a diffuse distribution of the parasites in all organs.

**Table 2:** Dimensions, nucleus characteristics and form proportions of *Mikrocytos* spp. according to sampling areas and years

Sample origin	Histology^a^				TEM^a^				
	No. of cells examined	Mean diameter (μm)	Diameter range (μm)	Eccentric nucleus (%)	No. of cells examined	Cell dimension range (μm)^b^	Nucleus dimension range (μm)^b^	Eccentric nucleus (%)	Dense form (%)
Quiberon 2008	200	1.91	1.08–3.2	80	20	2–4.72 × 1.31–4	0.94–2.22 × 0.56–2	75	80
Quiberon 2010	200	1.88	1.25–2.73	85	nd	nd	nd	nd	nd
Oléron 2010	200	2.01	1.19–3.01	71	nd	nd	nd	nd	nd
Audierne 2010	90	2.05	1.49–2.71	75	nd	nd	nd	nd	nd
Audierne 2011	200	1.79	1.16–2.94	76	20	1.83–3.2 × 1.07–2.42	0.68–1.38 × 0.59–1.09	85	100
Douarnenez 2011	200	2.15	1.28–3.17	87	12	2.03–3.79 × 1.60–2.50	0.73–1.70 × 0.71–1.26	75	75

^a^These observations were performed both on intracellular and extracellular parasites; the cell position of the parasites was not taken into account in the observations

^b^Length × width

*Abbreviation*: *nd* not done

The detection frequency of these microcell parasites varied between sampled areas and according the technics used (Table 1), but was relatively high everywhere except in Audierne Bay in 2010, where the sample was collected after the mortality outbreak. Histological analyses also revealed the presence of other organisms at low infection intensity (few parasites per individual), despite a high prevalence in some cases (Table 1). Specifically, some coccidian-like parasites were observed in the epithelia of the digestive gut or in the vesicular connective tissue surrounding it. Gregarine spores were noted in all tissues and rickettsia-like organisms were only detected in the epithelial cells of the digestive diverticula. Some trematode metacercariae were observed encysted in the connective tissue of the mantle. The presence of OsHV-1 virus was also detected in two samples from two different areas: one in 2010 from Quiberon Bay and another in 2011 from Audierne Bay. For both, the infection prevalence and intensity were low: six individuals infected in 2010 (< 91 OsHV-1 copies/μl) and three individuals in the 2011 sample (< 2 OsHV-1 copies/μl). Bacteriological analyses also revealed that only these two samples (Quiberon Bay 2010 and Audierne Bay 2011) presented bacterial colonies in abundance, whereas no dominant bacteria were observed in the other samples. Bacteria of the *Splendidus* clade were isolated from Quiberon Bay. For Audierne Bay, neither *Vibrio aestuarianus* strains nor bacteria of the *Splendidus* clade were isolated. The isolated strains were not characterized.

### Microcell characterization

Positive PCR results were only obtained with the primer pair Msp specific to the genus *Mikrocytos*, except on the samples from Audierne Bay, where a positive signal was also observed in some individuals with the primer pair MM (Table 1). Amplicons obtained with primer pairs Msp or MM were the same size as the *Mikrocytos mackini* amplified control for both primer pairs. All primer pair associations used in order to obtain a long *18S* fragment presented no positive signal in PCR (positive signal was observed for the positive control). Fifty-four individuals found by histology to be infected also presented a positive PCR signal with the primer pair Msp. In addition, 22 individuals were detected as infected by histology only and 12 by PCR only.

For each area and sampling date, 3 Msp PCR products were cloned and 3 clones for each PCR product were sequenced. In total, 36 putative *18S* rDNA sequences of 263 bp were obtained. These sequences shared 74.5–79% and 78–83% similarity with *Mikrocytos mackini* (HM563060.1) and *M. boweri* (KF297353), respectively. Clones obtained from the same PCR products did not necessarily have the same sequences and 16 distinct sequences could be identified and were submitted to GenBank (KY923792–KY923807). The alignment of these 16 sequences revealed point nucleotide substitutions, sometimes between clones obtained from the same PCR products. Additionally, sequences obtained from the Audierne Bay area presented major polymorphic regions in comparison with sequences from other areas (Additional file 1: Figure S1).

### Phylogenetic analyses of *18S* rDNA gene and ITS region

Pairwise comparison of the *18S* rDNA sequence of the microcells obtained from *Donax trunculus* with other closely related microcells suggested that the parasites from Audierne Bay were closer to parasites from Quiberon Bay, Douarnenez Bay or Oléron Island than to other groups of *Mikrocytos*, including *M. mackini*. The two French parasite groups had a mean genetic distance of 14.95% (Table 3).

**Table 3:** Estimates of evolutionary divergence (in %) over sequence pairs within the new species of *Mikrocytos* (for partial *18S* only) and between the *Mikrocytos* spp. The number of base substitutions per site obtained by averaging over all sequence pairs between groups is shown ± the standard error. Alignment length is specified for each gene region analysed

		*M. veneroïdes*	*M. donaxi*	*M. mackini*	*M. boweri*
Partial *18S* (287 nt)	*M. veneroïdes*	7.58 ± 3.94			
	*M. donaxi*	14.95 ± 4.18	7.5 ± 4.90		
	*M. mackini*	23.94 ± 9.09	26.46 ± 11.71		
	*M. boweri*	25.55 ± 9.62	21.52 ± 9.47	10.55% ± 3.19	
	*Mikrocytos* sp. Spain	79.54 ± 38.14	79.50 ± 38.47	62.29 ± 31.54	60.63 ± 28.32
*ITS1-5.8S-ITS2* (535 nt)	*M. veneroïdes*				
	*M. donaxi*	9.02 ± 2.03			
	*M. mackini*	12.33 ± 2.68	12.80 ± 2.65		
	*M. boweri*	12.11 ± 2.71	15.04 ± 3.31	11.10 ± 2.55	
	*Mikrocytos* sp. Spain	37.94 ± 7.81	33.14 ± 6.67	36.48 ± 7.48	34.59 ± 6.88
*ITS1* (186 nt)	*M. veneroïdes*				
	*M. donaxi*	6.67 ± 4.89			
	*M. mackini*	5.53 ± 4.18	6.79 ± 4.56		
	*M. boweri*	8.17 ± 5.75	14.58 ± 9.40	10.26 ± 6.86	
	*Mikrocytos* sp. Spain	20.26 ± 16.56	22.41 ± 17.77	18.79 ± 13.92	16.65 ± 12.20
*5.8S* (121 nt)	*M. veneroïdes*				
	*M. donaxi*	1.74 ± 1.20			
	*M. mackini*	3.57 ± 1.94	5.50 ± 2.48		
	*M. boweri*	1.76 ± 1.21	3.61 ± 1.86	2.66 ± 1.71	
	*Mikrocytos* sp. Spain	25.25 ± 54.05	22.21 ± 52.57	27.22 ± 55.08	27.20 ± 53.87
*ITS2* (223 nt)	*M. veneroïdes*				
	*M. donaxi*	13.16 ± 3.81			
	*M. mackini*	22.73 ± 6.26	19.34 ± 5.31		
	*M. boweri*	19.89 ± 5.11	16.38 ± 4.53	13.88 ± 4.08	
	*Mikrocytos* sp. Spain	33.40 ± 8.23	26.67 ± 6.85	33.34 ± 8.85	30.09 ± 7.69

**Fig. 5:**
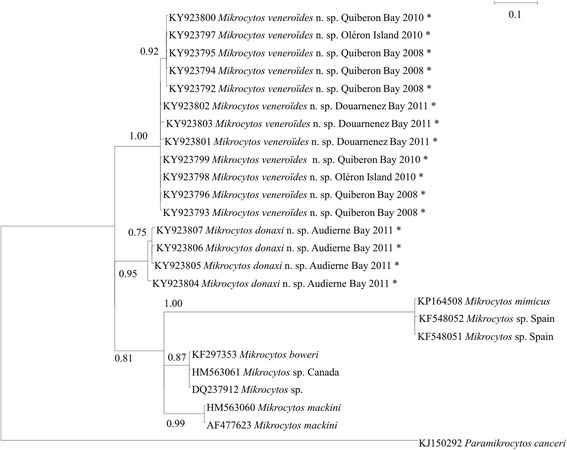
Phylogenetic tree (50% majority-rule consensus) using Bayesian Inference (MrBayes 3.1.2) based on the small subunit ribosomal gene of *Mikrocytos*. Numbers at the nodes are Bayesian posterior probabilities. *Paramikrocytos canceri* was used as the outgroup for *Mikrocytos* spp. based on the results of Hartikainen et al. [5]. Asterisks indicate sequences obtained in this study

The results obtained show that the parasites detected in the wedge clam in France are different from other *Mikrocytos* described. The phylogenetic analysis resulted in Bayesian posterior probabilities of 0.95 and 1.00 that support the existence of two distinct *Mikrocytos* species, one of which was only detected in Audierne Bay (Fig. 5).

In addition to the data obtained for the *18S* rRNA gene region, three individuals from Audierne Bay and three from Douarnenez Bay were selected for amplification using primers targeting the *ITS1-5.8S-ITS2* region. Six sequences of 370 bp were aligned, revealing two distinct sequences: one for parasites from Audierne Bay and one for parasites from Douarnenez Bay. These sequences showed 9.2 ± 2.03% divergence (Table 3) and were deposited in GenBank under the accession numbers KY923808 and KY923809, respectively.

**Fig. 6:**
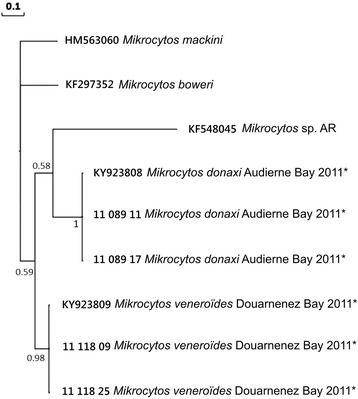
Phylogenetic tree (50% majority-rule consensus) using Bayesian Inference (MrBayes 3.1.2) based on the *ITS1-5.8S-ITS2* sequence array of *Mikrocytos*. Numbers at the nodes are Bayesian posterior probabilities. Asterisks indicate sequences obtained in this study

Phylogenetic analyses performed on the entire *ITS1-5.8S-ITS2* array confirmed the results obtained from the *18S* rRNA gene region, the existence of two distinct species of *Mikrocytos* in *Donax trunculus* in France. However, contrary to the analysis performed on the *18S* rRNA gene, the species detected in Audierne Bay appears distinct but closer to *Mikrocytos* sp. from Spain (KF548045) than from the other species in *D. trunculus* (Fig. 6).

Phylogenetic analyses were subsequently carried out for each region separately: *ITS1*, *5.8S* and *ITS2*. Depending on the target region, the relative position of both French *Mikrocytos* sequences in relation to other characterized *Mikrocytos* spp. was not the same (data not shown) and the genetic divergence between sequences was different (Table 3).

**Fig. 7:**
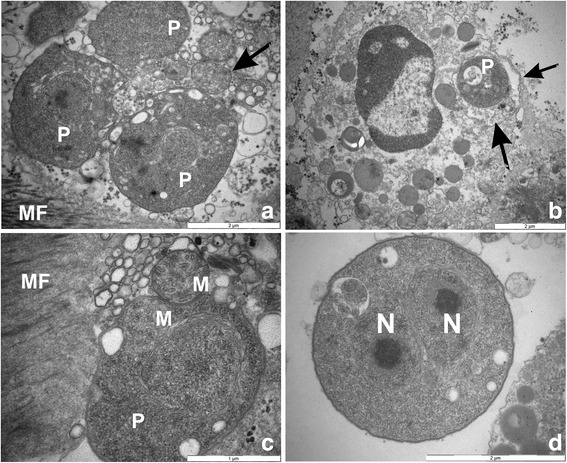
Transmission electron microscopy of *Mikrocytos veneroïdes* n. sp. infecting *Donax trunculus* mantle collected in Quiberon Bay. **a** Parasite inside a myocyte and near the myofibrils. Note the presence of mitochondria near the parasite cells (arrow). **b** Parasite in the cytoplasm of a haemocyte; note the presence of parasitophorous vacuole around the parasite (arrows). **c** Parasite near the myofibrils. Mitochondria tight against the surface of the parasite or inside the parasite cytoplasm; note the depression of parasite surface at the point of contact with mitochondria. **d** Binucleate stage of the parasite in an extracellular position; note the presence of the two nuclei. *Abbreviations*: M, mitochondria; MF, myofibrils; N, nucleus; P, parasite. *Scale-bars*: **a**, **b**, **d**, 2 μm; **c**, 1 μm

**Fig. 8:**
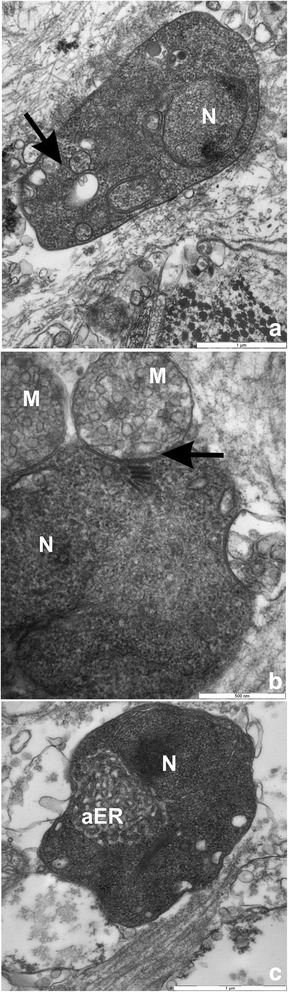
Transmission electron microscopy of *Mikrocytos donaxi* n. sp. infecting *Donax trunculus* mantle collected in Audierne Bay. **a** Dense form of the parasite with a very granulous cytoplasm and the presence of few large vesicles inside (arrow); note the eccentric position of the nucleus. **b** Parasite in tight contact with two mitochondria inducing a depression of the parasite membrane (arrow). **c** Parasite in the connective tissue at an endosomal stage, presenting a well-developed anastomosing endoplasmic reticulum near the nucleus. *Abbreviations*: aER, anastomosing endoplasmic reticulum; M, mitochondria; N, nucleus. *Scale-bars*: **a**, **c**, 1 μm; **b**, 0.5 μm

Based on morphological characteristics (see Table 2, Figs. 2, 3, 7 and 8), genetic sequences (Figs. 5 and 6) and the fact that these detections occurred in a new bivalve host species, the two *Donax* microcells detected in France were considered as two novel *Mikrocytos* species: the first one isolated from Oléron Island, Quiberon and Douarnenez Bays was named *Mikrocytos veneroïdes* n. sp. and the second one from Audierne Bay was named *Mikrocytos donaxi* n. sp.

**Family Mikrocytiidae Hartikainen, Stentiford, Bateman, Berney, Feist, Longshaw, Okamura, Stone, Ward, Wood & Bass, 2014**

**Genus** ***Mikrocytos*** **Farley, Wolf & Elston, 1988**

### *Mikrocytos veneroïdes* n. sp.

#### *Type-host*:

*Donax trunculus* Linnaeus, 1758 (Mollusca, Bivalvia, Heterodonta, Veneroida, Tellinoidea, Donacidae).

#### ***Type-locality***:

Atlantic coast of France: Oléron Island (45.50°N, 1.15°W), Quiberon Bay (47.54°N, 3.13°W) and Douarnenez Bay (48.11°N, 4.28°W).

#### ***Type-material***:

Fixed tissues (foot and mantle, accession No. 11/118/02, 11/118/10 and 11/118/21) infected with *Mikrocytos veneroïdes* n. sp. have been deposited at the OIE Reference Laboratory for infection with *Mikrocytos mackini*: Pacific Biological Station, Aquatic Animal Health Section, 3190 Hammond Bay Road, Nanaimo, British Columbia V9T 6 N7, Canada.

#### ***Representative DNA sequences***:

Nucleotide sequences of partial *18S* DNA region have been submitted in GenBank database under the accession numbers KY923792–KY923803 as well as a nucleotide sequence of the *ITS1-5.8S-ITS2* DNA region (accession number: KY923809).

#### ***ZooBank registration***:

To comply with the regulations set out in article 8.5 of the amended 2012 version of the International Code of Zoological Nomenclature (ICZN) [49], details of the new species have been submitted to ZooBank. The Life Science Identifier (LSID) of the article is urn:lsid:zoobank.org:pub:E1747954-CBDF-4F4A-BA22-E1C3CD1776BD. The LSID for the new name *Mikrocytos veneroïdes* is urn:lsid:zoobank.org:act:33B2B0F1-B94C-4744-A066-3037CB148276.

#### ***Etymology***:

The specific epithet refers to the order of the host *Donax trunculus*.

### Description

[Based on 32 specimens.] Spherical/ovoid parasites (Figs. 2 and 7) measuring 2.00–4.72 × 1.31–4.00 (2.73 ± 0.23 × 2.08 ± 0.18) μm, with a nucleus of 0.73–2.22 × 0.56–2.00 (1.26 ± 0.09 × 1.00 ± 0.08) μm (Table 2). Nucleus usually eccentric inside the parasite cell. Intracellular (within host cytoplasm cell) or extracellular parasites located in myocytes and vesicular cells of mantle, foot and adductor muscles (Fig. 2a) and to a lesser extent, in vesicular cells of digestive gland, gills and gonads. Occasionally, parasites inside neuronal ganglions and nerves (Fig. 2b) and haemocytes (Figs. 2c and 7b). In some tissues, especially in haemocytes, parasite presumably inside parasitophorous vacuole (Fig. 7b).No observation of mitochondria inside the parasite; however presence of parasite cells tight against host cell mitochondria and sometimes, depression of parasite surface at contact point (Fig. 7a, c).

No plasmodial or spore stage noted; several bi-nucleated cells in vesicular connective tissue and muscles present but no cytokinesis observed (Fig. 7d). Two main forms observed: dense form (predominant) and clear form. Dense form spheroid with eccentric nucleus and very granulous cytoplasm, giving it dark aspect. Clear form very similar to dense form, except that clearer cytoplasm despite strong granulation (Additional file 2: Figure S2).

### Remarks

The general morphological characteristics of *Mikrocytos veneroïdes* n. sp. were similar to those of other *Mikrocytos* spp.; its size, the absence of haplosporosomes and the absence of canonical mitochondria are in agreement with the different descriptions of *Mikrocytos* spp. [31, 50]. The close association of *M. veneroïdes* n. sp. with mitochondria was also one of the particularities of *Mikrocytos* spp. The three uninucleate parasite cell types (quiescent, endosomal and vesicular stage) described for *M. mackini* and *M. mimicus* [5, 50], could be observed in some samples of *M. veneroïdes* n. sp. The *M. veneroïdes* n. sp. quiescent cells were mainly observed in haemocytes and myocytes. They had a single nucleus with a granular nucleolus and, in their cytoplasm, the Golgi apparatus did not present budding. Some large uncoated vesicles were present in the cytoplasm. The endosomal cells presented a well-developed anastomosing endoplasmic reticulum, which could join the nuclear membrane to the cytoplasmic membrane. Finally, the vesicular cells presented large vesicles scattered in the cytoplasm and were frequent in myocytes. Some vesicular cells showed a dilatation of the nuclear membrane forming a cisternal chamber (Additional file 2: Figure S2). Meanwhile, the observation of these stages was occasional and not predominant in comparison to the clear and dense forms.

### *Mikrocytos donaxi* n. sp.

#### ***Type-host:***

*Donax trunculus* Linnaeus, 1758 (Mollusca, Bivalvia, Heterodonta, Veneroida, Tellinoidea, Donacidae).

#### ***Type-locality***:

Atlantic coast of France: Audierne Bay (47.85°N, 4.35°W).

#### ***Type-material:***

Fixed tissues (foot and mantle, accession No. 11/089/06, 11/089/25 and 11/089/27) infected with *Mikrocytos donaxi* n. sp. have been deposited at the OIE Reference Laboratory for infection with *Mikrocytos mackini*: Pacific Biological Station, Aquatic Animal Health Section, 3190 Hammond Bay Road, Nanaimo, British Columbia V9T 6 N7, Canada.

#### ***Representative DNA sequences:***

Nucleotide sequences of partial *18S* DNA region have been submitted in the GenBank database under the accession numbers KY923804–KY923807 as well as a nucleotide sequence of the *ITS1-5.8S-ITS2* DNA region (accession number: KY923808).

#### ***ZooBank registration:***

To comply with the regulations set out in article 8.5 of the amended 2012 version of the International Code of Zoological Nomenclature (ICZN) [49], details of the new species have been submitted to ZooBank. The Life Science Identifier (LSID) of the article is urn:lsid:zoobank.org:pub:E1747954-CBDF-4F4A-BA22-E1C3CD1776BD. The LSID for the new name *Mikrocytos donaxi* is urn:lsid:zoobank.org:act:307DFFED-D663-4ACA-A9AF-F98FC90B0896.

#### ***Etymology:***

The specific epithet refers to the genus of the host *Donax trunculus*.

### Description

[Based on 20 specimens.] Oval to round parasites (Fig. 3) measuring 1.84–3.20 × 1.07–2.42 (2.52 ± 0.18 × 1.76 ± 0.13) μm with a nucleus of 0.68–1.38 × 0.59–1.09 (1.15 ± 0.08 × 0.85 ± 0.07) μm (Table 2). Uninucleate cell with eccentric nucleus variable in form (Fig. 8a). Intracellular or extracellular parasite of myocytes and vesicular cells of mantle, foot, adductor muscles, nerves (Fig. 3b, c) and also, in vesicular cells of digestive gland, gills and gonads. No mitochondria noted inside parasite cytoplasm. Parasite cells often surrounded by mitochondria and sometimes, observation of depression of parasite membrane (Fig. 8b). No plasmodial or spore stage noted. Only dense form observed with very granulous cytoplasm (Fig. 8a).

### Remarks

*Mikrocytos donaxi* n. sp. presented the same morphological characteristics as *M. veneroïdes* n. sp. except for some particular traits. The nucleus of *M. donaxi* n. sp. seemed more frequent in eccentric position than in *M. veneroïdes* n. sp. Another difference in terms of size was also observed between these two species: *M. donaxi* n. sp. seemed smaller than *M. veneroïdes* n. sp. The close association with mitochondria was also noted for *M. donaxi* n. sp. as for *M. veneroïdes* n. sp. and for both parasites, in rare cases (observed in two parasite cells from Quiberon Bay and Audierne Bay), a mitochondrion appeared to be inside the cytoplasm of the microcell (Fig. 7c). *M. donaxi* n. sp. presented dense forms similar to those of *M. veneroides* n sp. but no clear forms were observed contrary to *M. veneroïdes* n. sp. The different uninucleate stages (quiescent, endosomal and vesicular cells) described for *Mikrocytos* spp*.* [5, 50] were not always observed in ultrastructure studies of *M. donaxi* n. sp.: in some samples, it was possible to distinguish some parasite cell types as the endosomal cells presenting a well-developed anastomosing endoplasmic reticulum (Fig. 8c).The non-observation of these different stages could be due to the dark aspect of the cytoplasm limiting ultrastructural observations of *M. donaxi* n. sp.

## Discussion

Infections with microcell parasites were observed in the four *Donax trunculus* wild beds and this is the first report of microcell parasites in this mollusc species. Results of morphological and molecular analyses indicated that these microcell parasites represent two new species of *Mikrocytos*: *Mikrocytos veneroïdes* n. sp. isolated from Oléron Island, Quiberon and Douarnenez Bays and *Mikrocytos donaxi* n. sp. from Audierne Bay. Although the general morphological characteristics of *Mikrocytos veneroïdes* n. sp. and *M. donaxi* n. sp. were similar to those of other *Mikrocytos* spp., few differences were noted between these two species. They generally had a nucleus in an eccentric position as in *M. mimicus* [5] and not in a central position as described for other *Mikrocytos* spp. [14–16, 18, 50]. Both for *M. donaxi* n. sp. and *M. veneroïdes* n. sp., different parasite forms were observed and these forms are consistent with those described in *M. mackini* [50] and *M. mimicus* [5]. Meanwhile, other forms were observed (dense and clear forms) as described for the oyster parasites, *Bonamia ostreae* and *B. exitiosa* Hine, Cochennec & Berthe, 2001 [51]: only dense forms were observed in *M. donaxi* n. sp. as in *B. ostreae* [2], whereas both dense and clear forms were observed in *M. veneroïdes* n. sp. as in *B. exitiosa* [52]. As described for *M. mackini*, there was a close association between *Mikrocytos* parasites and mitochondria [50] and in two cases, both for *M. donaxi* n. sp. and *M. veneroïdes* n. sp., mitochondria appeared to be inside the parasite cells. It was difficult, however, to determine whether these observations corresponded to a real mitochondria presence in the parasite cytoplasm or a sectioning artefact. Indeed, the cell membranes of the parasites were not well conserved and it was not possible to decide whether the mitochondria observed inside the cytoplasm were surrounded by a parasite cell membrane or not.

Phylogenetic analyses based on a 328 bp fragment of the *18S* gene showed that the microcells found in *D. trunculus* are distinct from other described *Mikrocytos* spp., including parasites previously reported in Europe, *M. mimicus* described in the Pacific oyster *Crassostrea gigas* in the UK and *Mikrocytos* sp. in *Ruditapes philippinarum* in Spain [5, 18].

Although the *18S* gene included some divergent domains, the average inter-specific and intraspecific sequence similarity was 87 and 98%, respectively [53]. Hence the maximum 85% sequence similarity observed here between *Mikrocytos donaxi* n. sp. and *M. veneroïdes* n. sp. and with other *Mikrocytos* species in the *18S* sequence is consistent with interspecific divergence levels. Mikrocytids are one of the most divergent eukaryotic lineages and have evolved at an extremely high rate, which might explain the lack of amplification using different primers designed for other *Mikrocytos* spp. in order to amplify a longer fragment of the *18S* gene [4, 5, 31]. The analysis of the *ITS1-5.8S–ITS2* array gave a different picture than the analysis of the *18S* gene fragment for *M. donaxi* n. sp., grouping this species with *Mikrocytos* sp. detected in Spain. Phylogenetic analyses carried out on each gene separately confirmed the existence of two distinct *Mikrocytos* species in *D. trunculus* in France but did not allow conclusively show their relative position in relation to other characterized *Mikrocytos* spp. *ITS1* and *ITS2* evolve faster than *18S* and *5.8S* and are usually more interesting to reveal intraspecific diversity. Additional sequence information, in particular longer *18S* gene sequence data, would help to solve the phylogenetic position of *M. donaxi* n. sp. and *M. veneroïdes* n. sp. in relation to the other molecularly characterized *Mikrocytos* spp.

In order to be able to detect potential co-infection, as observed for *Bonamia* parasites [54] or *Perkinsus* Levine, 1978 parasites [55], PCR products from the *18S* region were cloned and some clones were sequenced. No co-infection with *M. donaxi* n. sp. and *M. veneroïdes* n. sp. in any individual or in any area was observed, but different sequence types were detected in the same individual host for both *M. donaxi* n. sp. and *M. veneroïdes* n. sp. However, these sequence variations must be considered with caution because they may result from replication errors during cloning and it would be necessary to confirm them by performing direct sequencing on infected individuals.

*Mikrocytos donaxi* n. sp. and *M. veneroïdes* n. sp. were observed during significant *D. trunculus* mortality events in France. Questions on the pathogenicity of these parasites arise, because no unusual environmental conditions were noted during these mortality events. Some species of *Mikrocytos*, such as *M. mackini* or *M. mimicus*, are associated with mollusc mortalities [5, 10] and, in particular, the role of *M. mackini* in oyster mortality has been clearly demonstrated [8, 56]. However, some *Mikrocytos* spp., such as *M. boweri* or *Mikrocytos*-like form recorded in China, were detected in molluscs where no mortality has been observed [14, 16]. The *Mikrocytos*-like form described in clams from Spain was observed during mortality events, but its pathogenic role was not confirmed due to the presence of other potential clam pathogens [18]. In our study, the detection frequency of *M. donaxi* n. sp. and *M. veneroïdes* n. sp. was relatively high in the different areas studied (30–80%) whichever diagnostic technique was used, and no other known pathogen of *D. trunculus* was observed. Some parasites such as coccidian or gregarine spores were observed, but these pathogens are often observed in bivalve molluscs including in *D. trunculus* with no mortality observation [57]. Concerning the detection frequency, some differences appeared according the technique used, but these could be explained by either the analytical process (a small piece of tissue is used for PCR in comparison to histology) or the state of the animal (tissue necrosis may make it more difficult to observe parasites in tissues). In Audierne Bay, the detection frequency of *M. donaxi* n. sp. was low in 2010, in comparison to a high frequency in 2011 in *D. trunculus* from the same area. This low detection in 2010 could be explained by the fact that sampling was performed on alive animals after the mortality event, whereas in 2011 the sampling was done on moribund animals during the mortality event. For several mollusc pathogens, it is frequent to observe a prevalence peak just before mortality observation and a decrease of the parasite prevalence once the mortality has ended, which may explain our observations [58–60]. As for *M. mackini* or *M. mimicus* [5, 31, 61], a tissue necrosis was associated with the presence of parasite cells, suggesting a deleterious role of these parasites. The infected individuals generally presented a systemic infection. Our different observations suggest a pathogenic role of the new *Mikrocytos* spp. on *Donax trunculus*, but it will be necessary to confirm this role by experimental infections and field surveys.

Another question that remains unresolved concerns the origin of these parasites and, in particular, the detection of *Mikrocytos veneroïdes* n. sp. in different *D. trunculus* beds in a very short period of time. In our study, we demonstrated that *M. veneroïdes* n. sp. was already present in 2008 in one of the studied areas, but no information is available concerning the years prior or the other studied areas. The spread of this parasite by mollusc transfer is unlikely, because these clams are harvested and sold shortly after. No clam transfer exists between the different locations. Parasite transfers may be possible via infected fishing equipment since some fishermen have fishing licences for several *D. trunculus* beds. However, for two close areas that are frequently visited by fishermen (Douarnenez Bay and Audierne Bay), two different *Mikrocytos* species were detected, each species only located in one area. These parasites could have been present in these areas for a long time but at a low level of prevalence with their expression potentially dependent on different factors such as environmental conditions or clam density. Indeed, after these mortality events, the clam density dropped dramatically in the different wild beds and no mortality was reported the years after. Meanwhile, new mortality events occurred in most of the same areas in 2013 without a real increase in the clam density. As described for *M. mackini*, another important factor, the seawater temperature, could play a role in the disease appearance. For the disease development in oysters, *M. mackini* requires a seawater temperature below 10 °C for at least 3 months [62]. This characteristic was also observed for *M. mimicus* [5] and it also agreed with observed environmental conditions from *Mikrocytos* detection in China and in Canada [31]. In Europe, such conditions have been described in Spain and UK [5, 18]. In our study, no temperature data were available for the sampling areas but a study of clam populations and environmental conditions in the regional areas would be very interesting in order to know whether significant changes were observed, which might explain the detection of these parasites some years under favourable environmental conditions and their non-detection under unfavourable environmental conditions as observed in Quiberon Bay (*M. veneroïdes* n. sp. was detected in 2008, 2010 and 2013 but not in 2009 and 2011).

## Conclusions

*Mikrocytos veneroïdes* n. sp. and *M. donaxi* n. sp. represent two new species in the genus *Mikrocytos*, which until recently had been only composed of *M. mackini*. The detection of species of *Mikrocytos* in a new host also confirms the recent observation concerning the diversity of this genus. Nevertheless, further investigations are necessary to confirm the lineage of these *Mikrocytos* species, their pathogenic role and their disease epidemiology.

## Additional files

Additional file 1: Figure S1. Alignment of *Mikrocytos* small subunit ribosomal gene sequences obtained in the present study. (DOCX 197 kb)

Additional file 2: Figure S2. Transmission electron microscopy of *Mikrocytos veneroïdes* n. sp. at different stages. (DOCX 1814 kb)

## Abbreviations

ISH: in situ hybridization
PCR: polymerase chain reaction
REPAMO: REseau de PAthologie des Mollusques
RT: room temperature
TEM: transmission electron microscopy
