# Supplementary material for: Descriptions of Mikrocytos veneroïdes n. sp. and Mikrocytos donaxi n. sp. (Ascetosporea: Mikrocytida: Mikrocytiidae), detected during important mortality events of the wedge clam Donax trunculus Linnaeus (Veneroida: Donacidae), in France between 2008 and 2011
Source: Parasit Vectors. 2018 Mar 2;11:119. doi: 10.1186/s13071-018-2692-0 (PMC5834847; doi:10.1186/s13071-018-2692-0)
Supplement: Supplementary file 1 — Alignment of Mikrocytos small subunit ribosomal gene sequences obtained in the present study. (DOCX 197 kb) [file 13071_2018_2692_MOESM1_ESM.docx]

Alignments were performed using ClustalW included in MEGA 6 with default parameters and were manually checked for alignment gaps and missing data in nucleotide positions. The average length of the 16 SSU gene sequences in the final alignment file was 289 bp.

The alignment of these 16 sequences revealed point nucleotide substitutions, sometimes between clones obtained from the same PCR products. The correspondence between sample code and year and site is mentioned in Table S1.

For example, one clone from a wedge clam collected in Quiberon bay in 2008 has A instead of G in position 3 (Figure S1). Some substitutions concerned more than one sequence. For instance, three substitutions (T instead of C in position 88; C instead of T in position 90 and T instead of C in position 102) were observed in 5 distinct sequences obtained from wedge clams collected from Quiberon bay in 2008 and 2010 and from Oléron island (Figure S1).

Sequences obtained from the Audierne bay area presented major polymorphic regions in comparison with sequences from other areas and presented a few point substitutions, sometimes between clones obtained from the same animal (Figure S1).

Additional file 1: Table S1. Origin and description of SSU rRNA gene partial sequences obtained.

| **Parasite Name** | **Sequence names** | **Site** | **Year** | **Number of individuals (clones per individual)** | **Similarity between clones** | **Accession numbers** |
| --- | --- | --- | --- | --- | --- | --- |
| *Mikrocytos veneroïdes* | 08_131_02a  08_131_06a  08_131_17a  08_131_17b  08_131_17c | Quiberon | 2008 | 1 (3)  2 (3)  3 (3) | 1a = 1b = 1c  2a = 2b = 2c  3a  3b  3c | KY923792  KY923793  KY923794  KY923795  KY923796 |
| *Mikrocytos veneroïdes* | 10_138_02a  10_138_04a | Quiberon | 2010 | 1 (2)  2 (3)  3 (3) | 1a = 1b = 2a = 2b  2c = 3a = 3b = 3c | KY923799  KY923800 |
| *Mikrocytos veneroïdes* | 10_143_07a  10_143_07b | Oleron Island | 2010 | 1 (3)  2 (3)  3 (3) | 1a = 2a = 3a  1b = 1c = 2b = 2c = 3b = 3c | KY923797  KY923798 |
| *Mikrocytos donaxi* | 11_089_03a  11_089_10a  11_089_10b  11_089_3b | Audierne | 2011 | 1 (2)  2 (3)  3 (2) | 1a  2a  2b  1b = 2c = 3a = 3b | KY923804  KY923806  KY923807  KY923805 |
| *Mikrocytos veneroïdes* | 11_118_04a  11_118_10a  11_118_26a | Douarnenez | 2011 | 1 (1)  2 (1)  3 (1) | 1a  2a  3a | KY923801  KY923802  KY923803 |

Additional file 1: Figure S1. Alignment of *Mikrocytos* small subunit ribosomal gene sequences obtained in the present study (ClustalW in MEGA6.06).
