# Supplementary material for: Descriptions of Mikrocytos veneroïdes n. sp. and Mikrocytos donaxi n. sp. (Ascetosporea: Mikrocytida: Mikrocytiidae), detected during important mortality events of the wedge clam Donax trunculus Linnaeus (Veneroida: Donacidae), in France between 2008 and 2011
Source: Parasit Vectors. 2018 Mar 2;11:119. doi: 10.1186/s13071-018-2692-0 (PMC5834847; doi:10.1186/s13071-018-2692-0)

Additional file 2: Figure S2: Transmission electron microscopy of the *Mikrocytos veneroïdes* n. sp. parasites infecting *Donax trunculus* mantle collected in Quiberon bay. (A) Parasite in the connective tissue at an endosomal stage, presenting a well-developed anastomosing endoplasmic reticulum (aER) near the nucleus (N). Scale bar = 1µm (B) Clear form of the parasite in the connective tissue at a quiescent stage. Note the presence of few large vesicles in the cytoplasm (arrowhead) and the Golgi apparatus (arrow). Scale bar = 1µm (C) Dense form of the parasite in a myocyte at the vesicular stage presenting a cisternal chamber (arrow) and several large vesicles (arrowhead). Scale bar = 2µm.


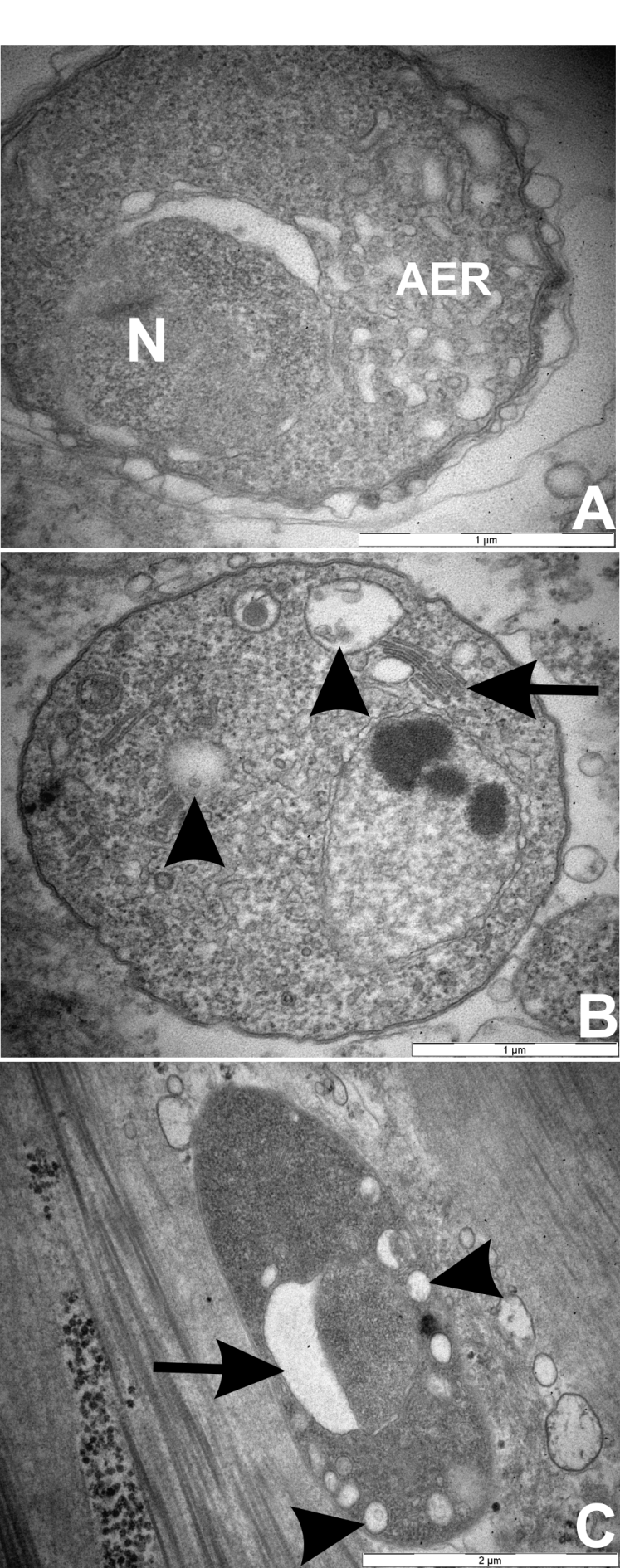

Supplement: Supplementary file 2 — Transmission electron microscopy of Mikrocytos veneroïdes n. sp. at different stages. (DOCX 1814 kb) [file 13071_2018_2692_MOESM2_ESM.docx]
